# Emergency Medicine Residents Experience Acute Stress While Working in the Emergency Department

**DOI:** 10.5811/westjem.2020.10.47641

**Published:** 2020-12-11

**Authors:** Adam J. Janicki, Stephanie O. Frisch, P. Daniel Patterson, Aaron Brown, Adam Frisch

**Affiliations:** * University of Pittsburgh, School of Medicine, Department of Emergency Medicine, Pittsburgh, Pennsylvania; † University of Pittsburgh, School of Nursing, Department of Acute and Tertiary Care, Pittsburgh, Pennsylvania

## Abstract

**Introduction:**

Acute stress may impair cognitive performance and multitasking, both vital in the practice of emergency medicine (EM). Previous research has demonstrated that board-certified emergency physicians experience physiologic stress while working clinically. We sought to determine whether EM residents have a similar stress response, and hypothesized that residents experience acute stress while working clinically.

**Methods:**

We performed a prospective observational study of physiologic stress including heart rate (HR), heart rate variability (HRV), and subjective stress in EM residents during clinical shifts in the emergency department. HR and HRV were measured via 3-lead Holter monitors and compared to baseline data obtained during weekly educational didactics. Subjective stress was assessed before and after clinical shifts via a Likert-scale questionnaire and written comments.

**Results:**

We enrolled 21 residents and acquired data from 40 shifts. Residents experienced an increase in mean HR of eight beats per minute (P < 0.001) and decrease in HRV of 53.9 milliseconds (P = 0.005) while working clinically. Subjective stress increased during clinical work (P <0.001). HRV was negatively correlated with subjective stress, but this did not reach statistical significance (P = 0.09).

**Conclusion:**

EM residents experience acute subjective and physiologic stress while working clinically. HR, HRV, and self-reported stress are feasible indicators to assess the acute stress response during residency training. These findings should be studied in a larger, more diverse cohort of residents and efforts made to identify characteristics that contribute to acute stress and to elicit targeted educational interventions to mitigate the acute stress response.

## INTRODUCTION

Medical education and clinical practice are stressful endeavors. High emotional and physiologic stress levels may impair cognitive performance and the ability to multitask, both of which are vital in the practice of Emergency Medicine (EM).^1^ The ways in which stress impacts performance are not completely understood as performance can be impaired, enhanced or unaffected under stressful conditions. Acute stress levels met by available resources may be perceived positively as a challenge, enhancing performance; however, those that outstrip available resources may be perceived as a threat and decrease performance.^1^,^2^,^3^,^4^

The task switching frequently required in the emergency department (ED) environment is particularly vulnerable to the effects of stress.^1^ In order to better understand the relationship between stress and performance in the ED, we must first determine if emergency physicians experience an acute stress response. Exposure to stressful situations and anticipation of a stressful situation contribute to changes in cardiac vagal tone with subsequent increases in heart rate (HR) and decreases in heart rate variability (HRV).^5^ It has previously been demonstrated that board-certified emergency physicians experience a decrease in HRV, a marker of physiologic stress, while working clinically.^6^ Unlike attending physicians, resident physicians have multiple additional available resources to use in the face of acute stress in the clinical environment, such as attending supervision and consultant services. It is unclear whether trainees have a stress response during clinical work and the effects of stress on the clinical learning environment remains unclear.

Residency training is commonly referred to as an extremely stressful endeavor. Previous research has demonstrated that, within simulated environments, resident physicians experience a stress response in the face of high-acuity simulation scenarios.^7^,^8^ Surgical, critical care, and medicine trainees experience stress as identified by a variety of subjective and physiologic measures while working clinically; however, few studies have assessed EM resident physician acute stress outside of the simulation environment.^9^–^14^ Mefford and associates demonstrated that EM residents experience a stress response while intubating patients in the operating room during an anesthesiology rotation; however, this is clinical environment is dissimilar to the emergency department.^14^ Additionally, while it has been recently demonstrated that EM residents experience an acute stress response while caring for critically ill patients, this patient population accounts for only a subset of ED patient volume.^15^

It is currently unclear whether EM resident physicians experience an acute stress response while working in the ED. Further, it is unclear whether chronic stress and burnout have an effect on residents’ acute stress response while working clinically. We hypothesized that EM resident physicians experience an acute physiologic stress response while working clinically.

## METHODS

### Study Design and Participants

We performed a prospective observational study evaluating surrogate markers of physiologic stress including HR and HRV in EM residents during clinical shifts with comparison to subjectively rated stress levels. The study was done at an urban, academic, Level I trauma center located in Pittsburgh, PA, with an annual census of over 55,000 patient visits from July 2018–December 2018. The study was approved by the institutional review board at the University of Pittsburgh Medical Center. All participation was voluntary and unpaid, and we obtained written informed consent.

Participants were a convenience sample of postgraduate year (PGY)-1 through PGY-3 EM residents. All residents working at the clinical site during the study period were eligible for enrollment. Exclusion criteria included history of cardiac arrythmia, cardioactive medication use, and pregnancy.

### Measurements

Demographic information, resident experience measured by the number of days in residency since enrollment, baseline chronic stress level, and burnout measurements were gathered during study enrollment. We assessed participant burnout via two 7-point Likert-scale measures previously validated in the assessment of resident physician burnout – one addressing emotional exhaustion and one depersonalization. The measure is based on the frequency with which participants experience various feelings or emotions with response options ranging from “Never” to “Daily.”^16^ Baseline chronic stress levels were assessed using the Perceived Stress Scale, one of the most widely used psychological instruments for measuring the perception of stress. It consists of 10 questions using a 5-point Likert scale (0 - Never, 4 – Very Often) to assess respondent’s feelings and thoughts during the prior month.^17^

**Population Health Research Capsule**

What do we already know about this issue?

High levels of acute stress may affect cognitive performance and task switching, both vital in Emergency Medicine (EM), in a variety of ways.

What was the research question?

Do EM residents experience an acute physiologic stress response as well as subjective stress while working clinically?

What was the major finding of the study?

EM residents experience acute subjective and physiologic stress while working clinically.

How does this improve population health?

Knowing that residents experience acute stress is the first step in understanding how stress impacts performance and how stress may affect patient care.

Previous research has demonstrated that HR and HRV are validated measures quantifying physiologic stress.^18^,^19^,^20^ While residents were working clinically, HR, detection of dysrhythmias, and HRV were measured via a 3-lead Holter monitor (Nasiff Associates, Inc. Central Square, NY) worn during the entire clinical shift. Clinical shifts were divided into three groups based on start times: morning (7 am and 11 am), evening (12 pm and 4 pm) and night (9 pm and 11 pm). Baseline HR and HRV were obtained via Holter measurements during weekly, dedicated educational didactic sessions, when residents are engaged in similar academic stimulation as clinical work. We used the standard deviation of all normal to normal R-R intervals (SDNN) measured in milliseconds (msec) to assess HR variability. The SDNN is the most commonly used measure of HRV and reflects autonomic influence on HRV.^21^,^22^ We collected all data within two weeks of enrollment to limit any potential changes in baseline stress, anxiety, and burnout.

Self-reported stress levels were determined before and after the shift via a single-item, 7-point Likert scale modified from a previously validated 10-point item in which participants responded to “What is your current stress level?” with answers ranging from not at all to extremely stressed.^23^ Each participant completed this self-assessment just prior to and after their clinical shift. In addition, at the conclusion of their shift residents were asked to “describe the most stressful part of [their] shift” in order to elicit qualitative data to inform future work.

We acquired ED census data and the number of patients evaluated by the resident physician from the electronic health record.

### Data Analysis

We assessed changes in HRV via a comparison from baseline in order to isolate individual-level changes. Subjective stress was assessed via the difference between pre- and post-shift scores.

We calculated descriptive statistics for participants’ HR, HRV, and self-reported stress levels. Paired t-test was used to compare baseline and on-shift HRV. We used analysis of variance (ANOVA) to compare physiologic data by PGY level and shift timing. As subjective data was nonparametric, Wilcoxon rank-sum test was used to compare pre- and post-shift subjective stress levels. We used Kruskal-Wallis ANOVA test to compare subjective stress by PGY level and shift timing.^24^ As HRV is a more specific indicator of acute stress than HR, multiple regression was used to assess the association between HRV and subjective stress levels, chronic baseline stress and burnout, ED census, resident experience, and patients per hour seen by the resident physician. We used analysis of residuals to confirm the assumptions of linearity. *P*-values of < 0.05 were considered statistically significant. All statistical analysis was performed using STATA 15.1 (Stata Corp, College Station, TX).

Qualitative data obtained was analyzed under the assumption that residents would be able to self-identify challenging components of their clinical work. Data were reviewed independently by the study investigators (AJ, AF) who subsequently met to discuss broad themes that emerged from responses. The study investigators discussed at length their independent findings and formulated themes by consensus. To evaluate for face validity, these themes were shared and reviewed with experts in graduate medical education who found the themes to be credible and interpretations accurate.

## RESULTS

Of the 23 eligible participants, 21 were included in the study. Two residents were excluded due to medication use. Our sample included 21 participants with a median age of 28 (interquartile range 26–30); four (19%) were women, and 17 (81%) men. There were six PGY-1, eight PGY-2, and seven PGY-3 level participants. Demographic and baseline stress, burnout, and callousness data are presented in Table 1.

Forty Holter monitor recordings were performed. Residents wore Holter monitors on three different shifts – 14 morning, 19 evening, and 7 overnight shifts. Mean daily ED census was 153 patients (95% confidence interval [CI], 149–157) and residents evaluated a mean of 1.5 patients per hour (95% CI, 1.4–1.7).

The mean baseline HR and SDNN for participants obtained at rest during weekly education didactics was 70 beats per minute and 262.8 msec, respectively. While working in the ED the mean HR was 78 beats per minute. One resident displayed findings concerning for tachyarrhythmia and was referred to cardiology for further evaluation. Data from these recordings were excluded from the analysis. The mean HRV measurements while working clinically was 208.9 msec. Residents experienced a statistically significant increase in mean HR (*P* < 0.001), maximum heart rate (*P* < 0.001), and decrease in HRV as measured by SDNN (*P* = 0.005) while working clinically. There was no difference in physiologic measures by PGY level (*P* = 0.11) or shift timing (*P* = 0.57).

Residents experienced a statistically significant increase in self-reported stress during clinical work (*P* < 0.001), decreasing by PGY level (*P* = 0.01). Overnight shifts caused less subjective acute stress when compared to morning or evening shifts. HRV measured by SDNN was negatively associated with subjective stress levels (r = 0.26, β = −0.76), indicating a correlation between increased physiologic acute stress and subjective acute stress, but this did not reach statistical significance (*P* = 0.09). Detailed comparisons of physiologic and subjective parameters are presented in Table 2.

Acute stress response measured by HRV via SDNN was not associated with resident experience, as measured by number of days in residency, (β = 61.7, *P* = 0.47), baseline chronic stress and anxiety (β = 1.69, *P* = 0.98), burnout (β = 0.37, *P* = 0.79), ED census (β = −1.67, *P* = 0.87), or the number of patients per hour seen by the resident physician (β = 0.2, *P* = 0.75). Daily ED census fluctuated around baseline levels.

Resident response to “describe the most stressful part of your shift” elicited multiple responses that were categorized into five broad themes. These findings are presented in Table 3.

## DISCUSSION

It is currently unclear how stress impacts performance. It can be impaired, enhanced or unaffected under stressful conditions.^1^–^4^ In order to better understand the complex relationship between stress and performance in EM trainees, we must first determine if they experience an acute stress response. We demonstrated that our cohort of EM resident physicians experience a physiologic acute stress response while working clinically as well as an increase in subjective stress levels. Although this physiologic stress response may have been correlated with subjective stress levels, this did not reach statistical significance. We suspect this is due to our relatively small sample size.

In our cohort, subjective stress levels decreased with rising PGY level, however we did not find that physiologic stress decreased significantly with increasing PGY level. This may be because experience affords some protection from the subjective stress response and not the physiologic response, but could also be due to our relatively small sample size, and thus more study is warranted. In addition, residents experienced less subjective stress on overnight shifts when compared to morning or evening shifts.

Prior research has demonstrated that non-EM trainees experience a physiologic stress response while performing high acuity procedures such as central venous access and emergency surgery in both simulated encounters and the clinical setting. Mefford and colleagues recently demonstrated that EM residents experience an acute stress response while intubating patients in the operating room.^14^ Additionally, EM trainees experience a physiologic stress response while performing medical resuscitations in a simulation environment and while caring for critically ill patients in the emergency department.^7^–^12^,^15^ Although these studies offer vital insight into resident stress, the intensive care unit, operating room, and simulation environment are far different clinical settings than that of an emergency physician in the ED setting. In addition to the physiologic stress response during high-acuity patient encounters during dedicated critical care shifts, residents may experience stress during low-acuity patient encounters with clinical situations outside of their control. Our narrative data suggest that residents often report an acute stress response in situations outside of their control such as social demands, inability to ensure a prompt admission, conflicting interests with colleagues, and inability to ensure resolution of symptoms and a prompt diagnosis. In a survey of EM residents, Perina and colleagues found that crowding, boarding, documentation, and ancillary support were top problems that resident identified as affecting their well-being.^25^ Given these findings, educational interventions that target managing the acute stress response itself may be beneficial. Additional research may better delineate settings in which stress improves performance and how this can be optimized in medical education.

It has been previously demonstrated that early high-stress encounters may result in significant amounts of stress being carried over into other patient encounters, even low-acuity ones.^26^ Further research in a larger cohort of resident physicians should be performed and more granular data obtained to better assess what is causing the acute stress response, if and how this stress affects performance, and how to best mitigate or manage the acute stress response.

Unlike prior work, our study evaluated both physiologic and subjective stress while working clinically the ED. It has been previously demonstrated that acute stress responses in the simulation setting and clinical setting are similar while caring for critically ill patients; however, much of emergency care in the United States also involves low-acuity and non-emergent presentations.^12^ Our study incorporated this low-acuity patient population and demonstrated similar acute physiologic changes and increases in subjective stress.^12^,^15^ Further, unlike other studies which examined stress at various time points during a clinical shift, we performed continuous HR and HRV monitoring throughout shifts in the hopes of capturing a more global assessment of physiological stress by incorporating the fluctuation in volume, patient acuity, and challenges experienced in most clinical shifts in the ED.^10^,^22^

## LIMITATIONS

While prior literature has shown heart rate and heart rate variability change during acute stress, their use as a proxy for stress is imperfect as physiologic parameters can be influenced by other sources such as activity or time of day. Although physical activity while on shift is normally limited to mild intensity walking, standing, and sitting, as evidenced by the absence of tachycardia in our HR data, prior work has demonstrated that any exercise will decrease HRV.^27^ Further, we assessed baseline HRV during residency didactic conference which takes place weekly from 8 am to 12 pm; thus, our HRV comparison may not take into account changes in HRV due to physical activity or shift time.

Our study was performed at a single institution and thus may reflect our specific clinical and training environment. Given our small sample size and relative homogeneity of our cohort, a larger study may reveal general trends regarding the specific resident, departmental, and shift characteristics that affect stress. It has been demonstrated that age and gender impact HRV, and therefore, our cohort of primarily male residents may have biased our results.^28^ Our small sample size limited our ability to stratify by multiple variables, including gender, age, and shift type. Further, although the instrument used to assess subjective stress was modified from tools with validity evidence and was assessed for content validity, it did not undergo additional validation testing. We did not assess subjective stress at fixed intervals throughout the shift which limited our ability to elicit specific clinical situations that provoke an acute stress response. We hoped that our qualitative data would help overcome this limitation by inferring themes for future study. We did not inquire about caffeine intake or other stimulants which may have affected subject HR; however, it has been previously demonstrated that caffeine does not have an effect on HRV or ventricular extrasystoles in our subject population of young adults.^29^ In addition, we did not specifically inquire about changes in at home life stressors. We hoped that evaluating subjective stress at the beginning of the shift would capture these acute changes, however the evaluation of physiologic stress did not take this into account.

## CONCLUSION

Emergency medicine resident physicians experience acute physiologic changes associated with stress as well as subjective acute stress while working clinically in the ED. EM trainees may experience stress during both high- and low-acuity patient encounters as well as in situations beyond their control. These findings should be studied in a larger, more diverse cohort of residents, and efforts should be made to identify resident, patient, and shift characteristics that contribute to the acute stress response and to determine the impact of acute stress on EM resident performance.

## Figures and Tables

**Table 1:** Participant demographics assessment (n = 21).

Age, years, median (interquartile range)	28 (27–28)
Gender, n (%)	
Male	17 (81)
Female	4 (19)
Relationship Status, n (%)	
Single	9 (43)
Married/civil partnership	12 (57)
Race, n (%)	
White	20 (95)
Black	1 (5)
Postgraduate year level, n (%)	
PGY-1	6 (29)
PGY-2	8 (38)
PGY-3	7 (33)
Resident experience level, days, mean (SD)	463.7 (279.2)
Baseline burnout, range 2–14, mean (SD)	6.6 (2.7)
Emotional exhaustion, range 1–7, mean (SD)	3.4 (1.2)
Depersonalization, range 1–7, mean (SD)	3.5 (2.0)
Perceived Stress Scale score, range 0–40, mean (SD)	13 (3.8)

*PGY*, post-graduate year; *SD*, standard deviation.

**Table 2:** Physiologic and subjective parameters of stress.

Physiologic parameters	Baseline	During clinical work	P-value
Heart rate, bpm^a^, mean (95% CI)	70 (67.8–73.2)	78 (74.7–81.7)	p < 0.001
Maximum heart rate, bpm^a^, mean (95% CI)	83 (78.4–86.7)	109 (103.6 – 113.8)	p < 0.001
Heart rate variability			
SDNN^b^, msec, mean (95% CI)	262.8 (230.8–294.7)	208.9 (184.9–232.8)	p = 0.005

Subjective parameters	Pre-shift	Post-shift	P-value

Subjective stress score, range 1–7, median (IQR)	2 (2–3)	4 (3–5)	p < 0.001
PGY level			
PGY 1	3 (2–3)	5 (5–5)	p = 0.01^c^
PGY 2	2 (2–3)	5 (2–5)	
PGY 3	2 (1–2)	3 (3–4)	
Shift timing			
Morning	2 (2–3)	5 (4–5)	p = 0.03^c^
Evening	2 (2–3)	5 (3–5)	
Overnight	2 (1–3)	2 (2–3)	

a beats per minute.

b standard deviation of all normal R-R intervals.

c Subjective stress levels compared using Kruskal-Wallis analysis of variance.

*CI*, confidence interval; *msec*, milliseconds; *IQR*, interquartile range; *PGY*, postgraduate year.

**Table 3:** Themes and examples from “describe the most stressful part of your shift.”

Theme	Examples
Demanding patients	“Patient who frequently presents demanding housing” “Patient unsatisfied by ED care” “Patient with chronic dental pain requesting pain medication” “Patient requesting narcotic pain medication in setting of chronic problem”
Inability to obtain a prompt admission and associated boarding	“Acute liver failure requiring ICU. Spoke with TICU, MICU, and SICU before finding a bed” “Patient was upset with length of stay due to bed availability” “Patient upset with length of stay”
Conflicting interests and disagreements with colleagues	“Dealing with difficult consultants” “Working out an admission between ENT and medicine”
Inability to ensure a diagnosis and symptom resolution	“Discharging patient home with ongoing neuro symptoms and no diagnosis” “Non-acute pathology I couldn’t do anything about”
High-acuity procedure	“Intubating a post arrest patient with a King airway in place” “Placing central line” “Placing an arterial line and a central line” “Trauma airway”

*ED*, emergency department; *ICU*, intensive care unit; *TICU*, trauma intensive care unit; *MICU*, medical intensive care unit; *SICU*, surgical intensive care unit.
